# Photocatalytic degradation of tetracycline hydrochloride with g-C_3_N_4_/Ag/AgBr composites

**DOI:** 10.3389/fchem.2022.1069816

**Published:** 2022-11-14

**Authors:** Jiahe Song, Kun Zhao, Xiangbin Yin, Ying Liu, Iltaf Khan, Shu-Yuan Liu

**Affiliations:** ^1^ Institute of Catalysis for Energy and Environment, College of Chemistry and Chemical Engineering, Shenyang Normal University, Shenyang, China; ^2^ School of Environmental and Chemical Engineering, Jiangsu University of Science and Technology, Zhenjiang, China; ^3^ Department of Pharmacology, Shenyang Medical College, Shenyang, China

**Keywords:** G-C_3_N_4_, Ag/AgBr, Z-scheme heterojunction, photocatalytic degradation, tetracycline hydrochloride

## Abstract

Graphite carbon nitride (g-C_3_N_4_), as a polymer semiconductor photocatalyst, is widely used in the treatment of photocatalytic environmental pollution. In this work, a Z-scheme g-C_3_N_4_/Ag/AgBr heterojunction photocatalyst was prepared based on the preparation of a g-C_3_N_4_-based heterojunction *via in-situ* loading through photoreduction method. The g-C_3_N_4_/Ag/AgBr composite showed an excellent photocatalytic performance in the degradation of tetracycline hydrochloride pollutants. Among the prepared samples, g-C_3_N_4_/Ag/AgBr-8% showed the best photocatalytic ability for the degradation of tetracycline hydrochloride, whose photocatalytic degradation kinetic constant was 0.02764 min^−1^, which was 9.8 times that of g-C_3_N_4_, 2.4 times that of AgBr, and 1.9 times that of Ag/AgBr. In the photocatalytic process, ^•^O^2–^ and ^•^OH are main active oxygen species involved in the degradation of organic pollutants. The photocatalytic mechanism of g-C_3_N_4_/Ag/AgBr is mainly through the formation of Z-scheme heterojunctions, which not only effectively improves the separation efficiency of photogenerated electron-hole pairs, but also maintains the oxidation and reduction capability of AgBr and g-C_3_N_4_, respectively.

## Introduction

With the rapid development of social economy and the chemical industry, all kinds of pollutants, such as heavy metal ions, organic pesticides, chemical dyes, medical residues and other pollutants, are discharged into water bodies in large quantities and participate in water cycle, leading to the deterioration of water environment, which has posed a huge threat to human life and health as well as the survival of various aquatic organisms. In the pharmaceutical industry, with an increasing demand for drugs, especially antibiotics, antibiotics have begun to be widely used worldwide. Antibiotics cannot be completely absorbed after entering human or animal bodies, leading the unabsorbed antibiotics to be discharged into the water environment as metabolites, which are even in their original state with metabolic activities. Antibiotics of low doses discharged into the environment for a long time will lead to an enhanced drug resistance for sensitive bacteria. Moreover, drug-resistance genes can expand and evolve in the environment, posing a potential threat to the ecological environment and human health. In addition to causing bacterial resistance, antibiotics may also produce certain toxicity to other organisms. Therefore, as new micro-pollutants, antibiotics have characteristics including a low concentration, a high toxicity and a difficult treatment in the environment.

Traditional water treatment technologies mainly include physical, chemical and biological methods. Physical methods refer to enriching and treating pollutants in turn by physical means, which have characteristics including relatively simple operation and a low cost, but they are easy to cause secondary pollution (Qi et al., 2022). Chemical methods refer to water treatment methods through which organic pollutants are oxidized by adding strong oxidizing chemicals. However, in practical application, a high use of oxidants is required, which may cause secondary pollution very easily due to incomplete reactions. Biological methods refer to further transforming organic matters in water into nutrients or other small molecular substances required by microorganisms through the metabolism of microorganisms. The process of microbial methods is simple, through which secondary pollution can be avoided. However, a microbial treatment cycle is long and the microbial activity is greatly affected by the outside world, which is not conducive to the treatment of organic wastewater that is difficult to be biodegraded due to the changeable environment. Therefore, it is urgent to find a new water pollution treatment technology with a high efficiency, a low cost and less secondary pollution.

In photocatalysis technology, solar energy is used to drive and excite light catalysts to produce a variety of strong-oxidizing active substances, destroy the molecular structure of pollutants, and finally convert organic pollutants into CO_2_, H_2_O or other pollution-free small molecules, which can be directly discharged into the environment (Wang et al., 2019; Li et al., 2020b; Li et al., 2022b; Wang L. et al., 2022). Compared with traditional water pollution treatment methods, the photocatalytic degradation method has advantages including a high efficiency, simplicity, a good reproducibility and an easy treatment, which is often used to degrade organic pollutants (Li et al., 2022a; Tao et al., 2022; Wang W. et al., 2022). The core of photocatalysis technology is the development of new photocatalysts (Yang et al., 2021; Wang et al., 2022c; Wang et al., 2022e). At present, traditional semiconductor photocatalyst materials are mainly inorganic compounds, but their large-scale application has been restricted due to limited resources.

Graphite like carbon nitride (g-C_3_N_4_) is a polymeric semiconductor photocatalyst material. Compared with traditional photocatalysts such as metal oxides, metal sulfides and metal halides, g-C_3_N_4_ is a non-metallic semiconductor material with a narrow band gap of ∼2.7 eV, which has attracted extensive attention due to its unique optical and electronic properties, a high controllability, a good chemical stability, non-toxicity and other characteristics. A large number of studies have shown that g-C_3_N_4_ is an ideal photocatalyst material in terms of the photocatalytic treatment of water and air pollution as well as hydrogen production, etc (Li et al., 2020a; Xiong et al., 2020; Li et al., 2021; Wang et al., 2022b). However, g-C_3_N_4_ itself has some shortcomings, especially its small specific surface area and high photogenerated electron-hole recombination rate, which reduce its photocatalytic efficiency and utilization meanwhile limiting its industrial application (Wang et al., 2022a; Li et al., 2022c; Wang et al., 2022d). Therefore, the research on g-C_3_N_4_ to improve its photocatalytic activity has become a research hotspot.

To improve the photocatalytic activity of g-C_3_N_4_, researchers usually modify existing photocatalysts. Nowadays, there are several ways to modify them, including element doping, noble metal modification, semiconductor recombination or improving the photocatalytic performance of materials by changing their microstructure. Therefore, the structure and physicochemical properties of g-C_3_N_4_ can be changed through defect regulation, surface noble metal modification and semiconductor material recombination, so that it can have a wider visible light absorption range, its electron-hole recombination efficiency is reduced and its photocatalytic activity is improved.

In this study, silver (Ag), a noble metal matching the Fermi level of g-C_3_N_4_, was selected to modify g-C_3_N_4_, change its energy band structure and form a plasma effect. At the same time, semiconductor materials AgBr and g-C_3_N_4_ were compounded to form a Z-type heterostructure, whose purpose was to enhance the light absorption capacity of photocatalysts, improve the separation efficiency of photogenerated carriers and enhance the redox capacity as well as activity of photocatalysts. The photocatalytic activity of the photocatalysts obtained was investigated through the simulated solar light degradation of tetracycline hydrochloride with the g-C_3_N_4_/Ag/AgBr composites prepared. The charge separation and migration behavior of the samples prepared were studied through different characterization and analysis methods. The possible enhancement mechanism of photocatalysis was reasonably described in combination with the results of a free radical capture experiment based on the semiconductor energy band theory.

## Experimental section

### Preparation

The g-C_3_N_4_ catalyst is prepared by placing melamine in a crucible and firing it in a muffle furnace at 550°C for 4 h, then the samples obtained are grinded and collected after cooling. To load different amounts of Ag/AgBr on the surface of g-C_3_N_4_ and produce g-C_3_N_4_/Ag/AgBr composites, g-C_3_N_4_-Ag/AgBr photocatalyst was prepared through simple *in-situ* coprecipitation method and photo-reduction-assisted method. The specific steps are as follows: 0.2 g of g-C_3_N_4_ is dispersed in 60 ml of deionized water to obtain Solution A. Then AgNO_3_ (0.1 mol/L) solutions of different volumes are added to Solution A respectively, which are continuously stirred for 30 min. Then, drop KBr (0.1 mol/L) solution into the above solutions respectively, and continue stirring in a dark condition for 3 h. After this step is completed, irradiate the solution under a xenon lamp for 1 h, and finally naturally cool the whole system to room temperature. After centrifugation, wash the solution with anhydrous ethanol and deionized water for three times alternately. Dry the collected samples in an oven at 70°C for 12 h, and finally grind and collect them. By changing the volume of AgNO_3_ solution added, g-C_3_N_4_/Ag/AgBr composites loaded with 4 wt%, 6 wt%, 8 wt%, and 10 wt% of Ag can be obtained, which are labeled as g-C_3_N_4_/Ag/AgBr-4%, g-C_3_N_4_/Ag/AgBr-6%, g-C_3_N_4_/Ag/AgBr-8%, and g-C_3_N_4_/Ag/AgBr-10% respectively. AgBr and Ag/AgBr monomers are prepared through the same preparation process as above without adding g-C_3_N_4_.

### Characterization

The crystal structure of the samples was obtained *via* an XRD diffractometer produced by Bruker D8, Germany, with a scanning range of 10°–80°. TEM and HRTEM photos were obtained through transmission electron microscopy (JEOL 2100). For a Fourier transform infrared (FTIR) analysis, a Nicolet Magna 560 spectrophotometer (US) was used. An ESCALAB MKII X-ray photoelectron spectrometer (UK) and Mg-K *α* were used. The binding energy and element state of composite materials were measured through radiation. The ultraviolet-visible absorption spectrum (UV-vis DRS) of the samples at the wavelength of 200–800 nm was measured by a spectrophotometer with an integrating sphere (Hitachi, U-4100), with BaSO_4_ as the reference.

### Photocatalytic activity test

The photocatalytic activity of g-C_3_N_4_/Ag/AgBr composite in the degradation of tetracycline hydrochloride is measured at room temperature. The specific experimental steps are as follows: before the photochemical reaction, 50 ml of tetracycline hydrochloride solution (20 mg/L) and 32 mg of g-C_3_N_4_/Ag/AgBr composite photocatalyst were added and stirred in the dark for 1 h to achieve an adsorption-desorption equilibrium. Then the reaction mixture was irradiated with an iodine tungsten lamp to induce the occurrence of photocatalytic reaction. During the reaction, 3 ml of reaction solution was extracted from the reaction system every 20 min, which was added to the UV cuvette after centrifugation and filtration. The absorbance was measured by a UV-visible spectrophotometer. The concentration of tetracycline hydrochloride was determined at its maximum absorption wavelength of 356 nm by an ultraviolet spectrophotometer.

## Results and discussion

### XRD

**FIGURE 1:**
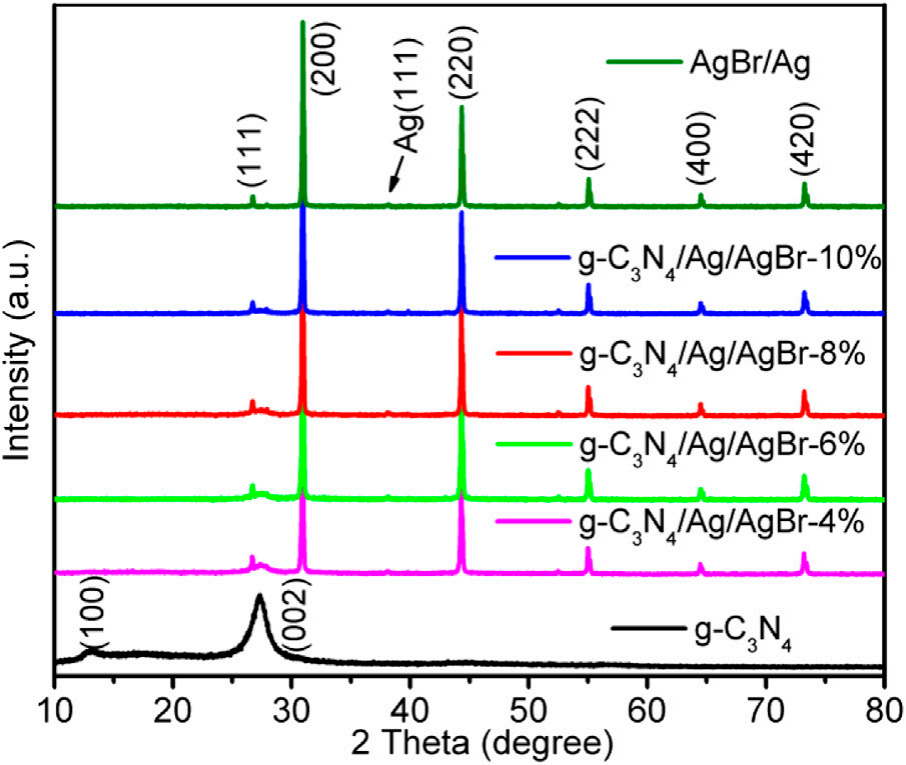
XRD pattern of g-C_3_N_4_, Ag/AgBr, and g-C_3_N_4_/Ag/AgBr.

The composition and crystal phase of the g-C_3_N_4_/Ag/AgBr composite are analyzed through XRD measurement. The XRD diffraction spectra of each sample are shown in Figure 1. Among them, the diffraction peak at 13.1° corresponds to crystal plane (100) of g-C_3_N_4_, representing a regular arrangement of 3-s-triazine ring units on the g-C_3_N_4_ plane; the diffraction peak at about 27.5° belongs to crystal plane (002) of g-C_3_N_4_, representing the layer spacing among g-C_3_N_4_ layers (Qi et al., 2020a). The main XRD diffraction peaks of Ag/AgBr appear at 26.7°, 31.0°, 44.3°, 55.0°, 64.5°, and 73.3°, whose corresponding crystal planes are plane (111), (200), (220), (222), (400), and (420) of AgBr (JCPDS: 06-0438) (Chen et al., 2020). In addition, a weak diffraction peak can also be observed at 38.1°, which corresponds to plane 111) of Ag (JCPDS: 04-0783) (Li S. et al., 2020). The characteristic peaks of g-C_3_N_4_ and AgBr can be observed in the XRD diffraction pattern of the g-C_3_N_4_/Ag/AgBr composite, indicating the existence of g-C_3_N_4_ and AgBr in the composite. In addition, the peaks of Ag can also be observed. However, due to the low content and uniform dispersion of Ag in the system, the diffraction peak intensity is weak.

### Fourier transform infrared

**FIGURE 2:**
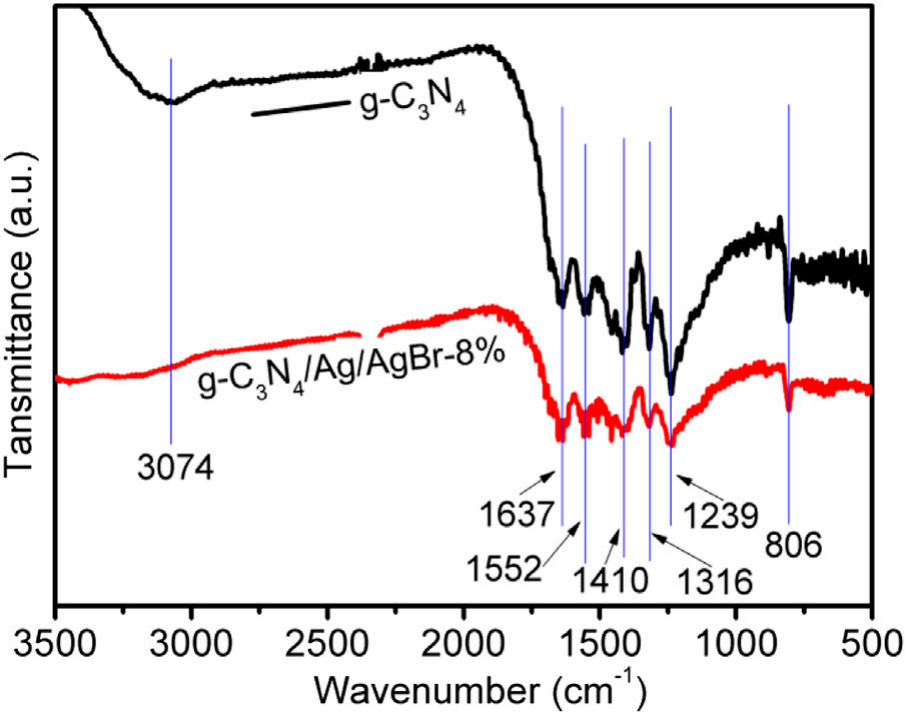
FTIR spectrum of g-C_3_N_4_ and g-C_3_N_4_/Ag/AgBr-8%.

The chemical bonds and functional group composition of g-C_3_N_4_/Ag/AgBr-8% and g-C_3_N_4_ were studied *via* FTIR measurement, as is shown in Figure 2. The absorption peak between 1239 and 1637 cm^−1^ in the g-C_3_N_4_ spectrum corresponds to the typical C (sp^2^) = N and C (sp^2^)-N stretching vibration (Qi et al., 2020b). The wider absorption peak at 3,074 cm^−1^ is attributed to the N-H stretching vibration of g-C_3_N_4_ or the O-H stretching vibration of adsorbed water (Qi et al., 2021). In addition, the peak at 806 cm^−1^ corresponds to the vibration mode absorption band of triazine (Zhao et al., 2020). It can be seen from the spectrum that the absorption peak of g-C_3_N_4_/Ag/AgBr-8% is similar to the characteristic absorption peak of g-C_3_N_4_. When Ag/AgBr is compounded with g-C_3_N_4_, the intensity of the absorption peak of the g-C_3_N_4_/Ag/AgBr sample becomes weak, indicating the successful preparation of the composite photocatalyst.

### Ultraviolet-visible diffuse reflectance spectroscopy

**FIGURE 3:**
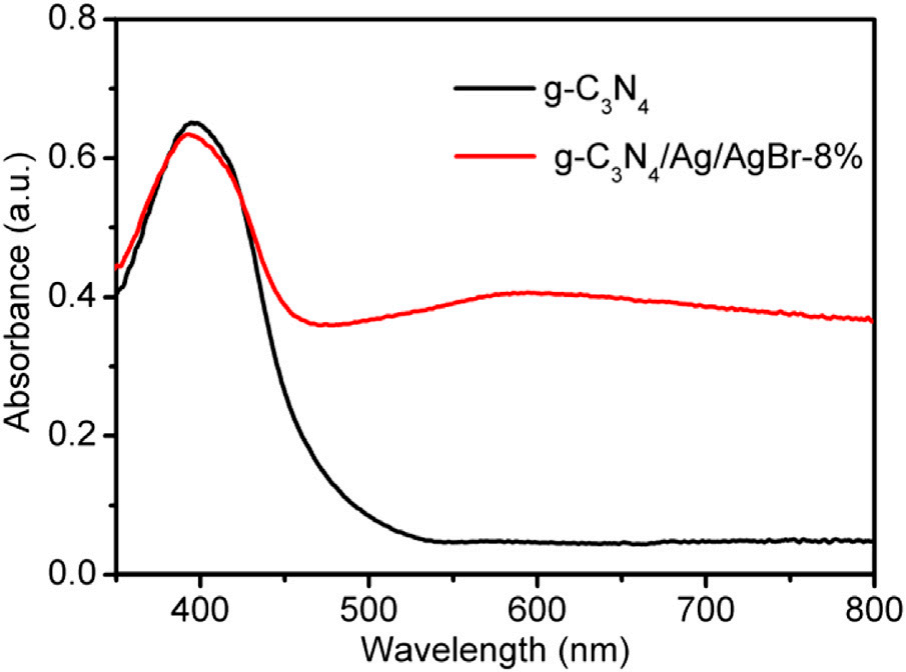
UV–vis DRS spectrum of g-C_3_N_4_ and g-C_3_N_4_/Ag/AgBr-8%.

The optical absorption property of samples was analyzed *via* ultraviolet-visible diffuse reflectance spectroscopy (UV-DRS). The UV-DRS spectrum of g-C_3_N_4_ and g-C_3_N_4_/Ag/AgBr-8% is shown in Figure 3, whose light absorption edge is located at about 456 nm and 480 nm. Compared with g-C_3_N_4_, the absorption band of the g-C_3_N_4_/Ag/AgBr-8% composite is red shifted, its visible light absorption range is increased, and the energy required for its transition is reduced, which is due to the introduction of Ag/AgBr. A stronger absorption is also shown in the whole wavelength range of visible light, which is also attributed to the surface adsorption of Ag/AgBr nanoparticles.

### TEM

**FIGURE 4:**
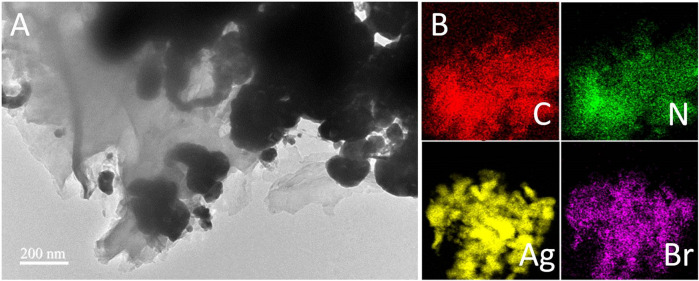
**(A)** TEM image of g-C_3_N_4_/Ag/AgBr-8% and **(B)** corresponding elemental mapping of C, N, Ag, and Br.

To further identify the microstructure of the g-C_3_N_4_/Ag/AgBr-8% composite in detail, the material was characterized through TEM measurement. Figure 4A shows the images of the local sheet structure of g-C_3_N_4_/Ag/AgBr-8%, where Ag/AgBr can be observed on the surface of the sheet. In addition, the nanoparticles formed by the accumulation of reduced Ag can also be observed. EDS technology was used to study the composition and spatial distribution of elements in the sample. Figure 4B shows the distribution of elements C, N, Ag, and Br, which are uniformly distributed on the whole composite, indicating that AgBr is successfully and uniformly loaded on the surface of g-C_3_N_4_. The above TEM results show that g-C_3_N_4_, Ag, and AgBr are in close contact with each other, and a heterojunction structure is formed between each two components, which is conducive to the separation and transfer of photogenerated charges, and is also one of the main reasons for the enhanced photocatalytic activity.

### XPS

**FIGURE 5:**
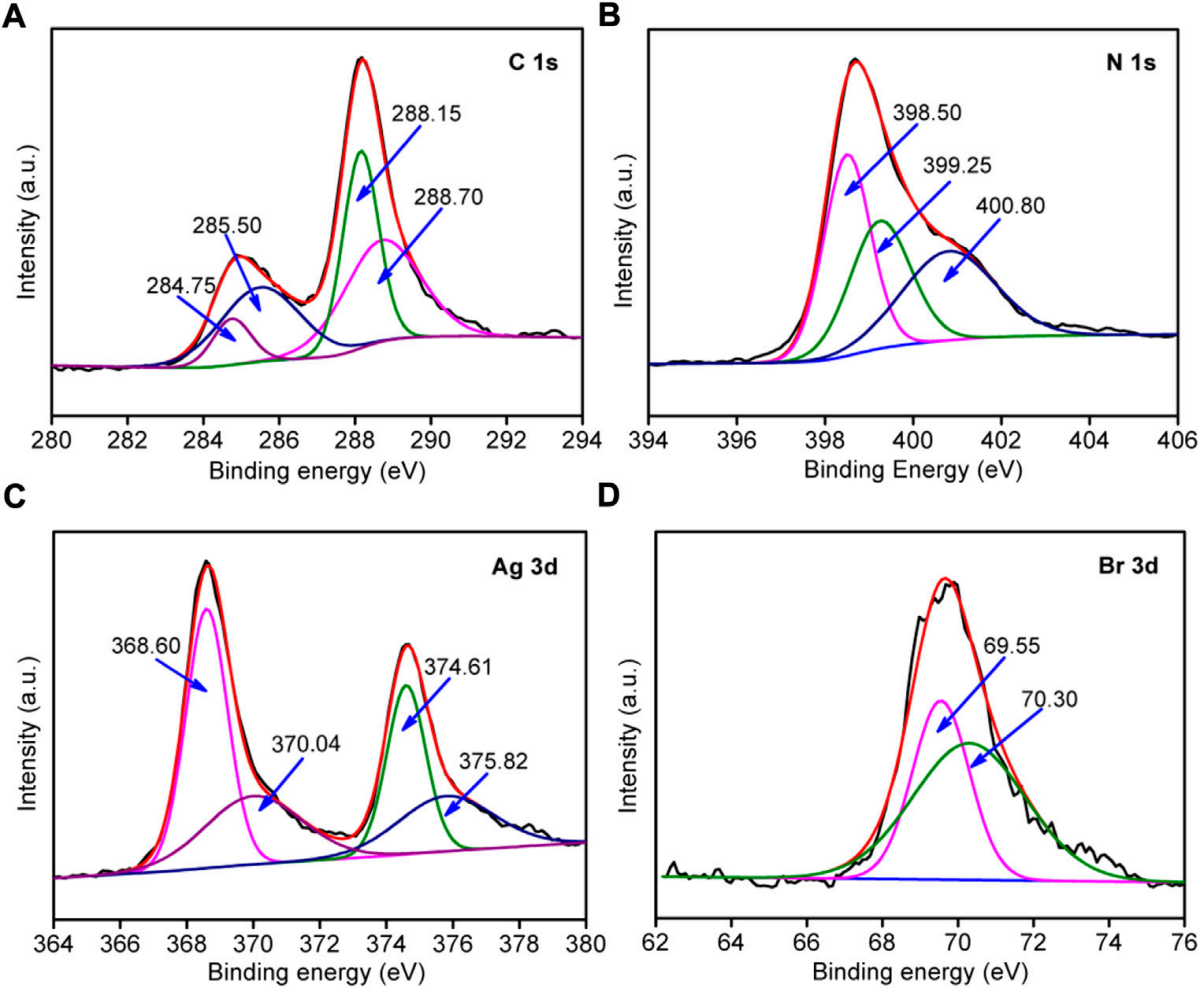
High-resolution XPS spectra of g-C_3_N_4_/Ag/AgBr-8% for C 2p **(A)**, N2p **(B)**, Br 1s **(C)**, and Ag 3d **(D)**.

The chemical composition and element valence changes in g-C_3_N_4_/Ag/AgBr-8% composite were analyzed *via* XPS measurement, as is shown in Figure 5. Figure 5A shows that the high-resolution XPS spectrum of C 1s can be divided into four peaks with a binding energy of 284.75, 285.50, 288.15, and 288.70 eV. The peak is located at 284.75 eV, which corresponds to the adventitious surface carbon (Qi et al., 2019a). The peak at 285.50 eV is a C-C bond group (Qi et al., 2020c). The peaks at 288.15 and 288.70 eV are an assigned N-C=N bond and C-(N)_3_ respectively (Liu et al., 2022). Figure 5B shows that the N1s spectrum can be divided into three peaks with a binding energy of 398.50, 399.25, and 400.80 eV respectively. The peak with a binding energy of 398.50 eV corresponds to the C-N=C bond of the 3-s-triazine ring, and the peaks at 399.25 as well as 400.80 eV belong to the C-(N)_3_ and N-H structure respectively (Zhao et al., 2021). Figure 5C shows the high-resolution energy spectrum of Ag 3d, and four peaks with different positions can be obtained through further fitting. The peaks with a binding energy of 368.60 eV and 374.61 eV belong to Ag^+^ 3d_5/2_ and Ag^+^ 3d_3/2_ in AgBr. The peaks with a binding energy of 370.04 eV and 375.82 eV belong to Ag^0^ 3d_5/2_ and Ag^0^ 3d_3/2_, indicating the existence of Ag simple substances (Qi et al., 2019b). As is shown in Figure 5D, there are peaks of Br 3d_5/2_ and Br 3d_3/2_ at 69.55 and 70.30 eV in the binding energy of Br 3d, indicating that Br element exists in the system with a negative valence (Zhang et al., 2020).

### Photoluminescence and photocurrent analysis

**FIGURE 6:**
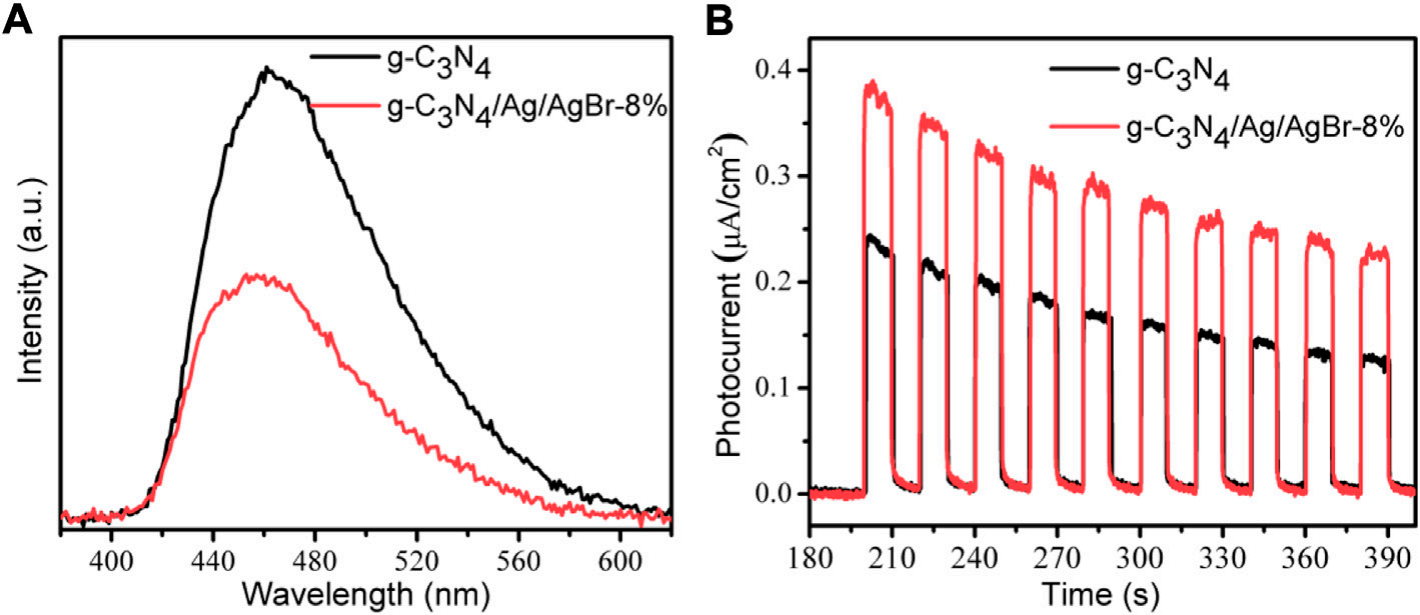
PL spectrum **(A)** and photocurrent response **(B)** of g-C_3_N_4_ and g-C_3_N_4_/Ag/AgBr-8%.

Photoluminescence spectra (PL) can reflect the transport efficiency of a carrier to some extent. Generally, the higher the recombination rate of photogenerated carriers is, the stronger the corresponding fluorescence intensity will be. The fluorescence spectrum of g-C_3_N_4_ and g-C_3_N_4_/Ag/AgBr-8% composite was characterized, whose results were obtained, as is shown in Figure 6A. Compared with g-C_3_N_4_, the PL intensity of g-C_3_N_4_/Ag/AgBr-8% is weaker, which indicates that the loading of Ag/AgBr on g-C_3_N_4_ is conducive to the separation of photogenerated carriers, which will help to improve the photocatalytic activity.

Photocurrent density is an effective means to reveal the separation and transmission characteristics of photogenerated electrons in semiconductor materials. The higher the photocurrent density is, the higher the separation efficiency of photogenerated carriers is. Through the electrode preparation method, g-C_3_N_4_ and g-C_3_N_4_/Ag/AgBr-8% composite was prepared into corresponding working electrodes, and a transient photocurrent cycle test was carried out in light and dark conditions. The photocurrent results are shown in Figure 6B. Both the two materials can generate photocurrent under simulated solar light irradiation, indicating that they can be excited to generate photogenerated carriers under simulated solar light irradiation. The photocurrent intensity produced by g-C_3_N_4_/Ag/AgBr-8% (∼0.28 μA/cm^2^) is higher than that produced by g-C_3_N_4_ (∼0.16 μA/cm^2^), indicating that the separation effect of photogenerated electron-hole pairs is enhanced by constructing heterojunctions between g-C_3_N_4_ and Ag/AgBr.

### Photocatalytic activity

**FIGURE 7:**
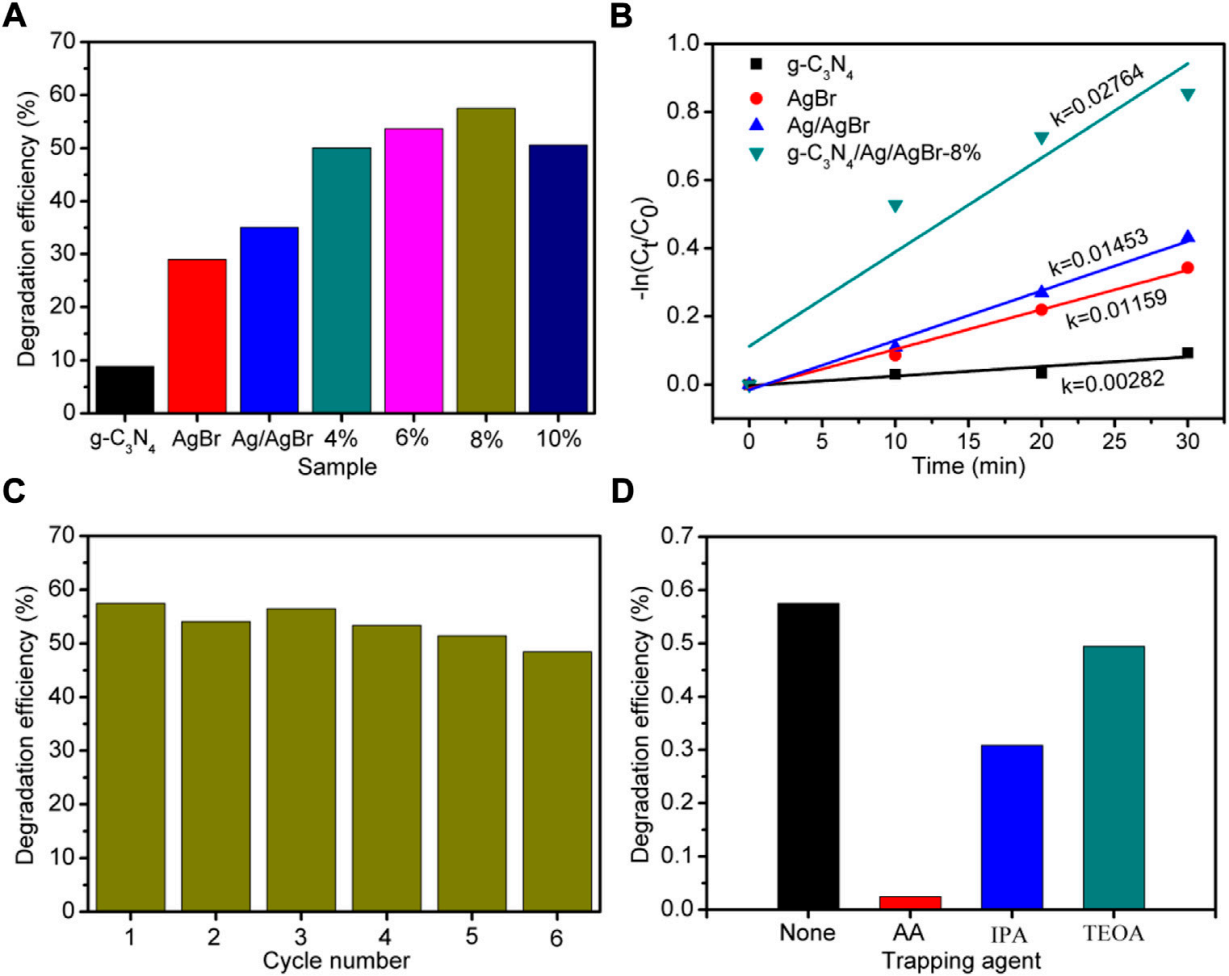
**(A)** Photodegradation efficiency of tetracycline hydrochloride using g-C_3_N_4_, AgBr, Ag/AgBr, and g-C_3_N_4_/Ag/AgBr as photocatalysts in 30 min. Here x% represents the sample of g-C_3_N_4_/Ag/AgBr-x%. **(B)** Reaction rate curve, **(C)** recycling test and **(D)** free radical capture experiment on g-C_3_N_4_/Ag/AgBr-8%.

To explore the effect of g-C_3_N_4_/Ag/AgBr composite on improving the photocatalytic activity under simulated solar light irradiation, the g-C_3_N_4_/Ag/AgBr composite prepared was used to conduct a degradation experiment on tetracycline hydrochloride solution under simulated sunlight irradiation. Figure 7A shows the photodegradation efficiency of tetracycline hydrochloride using g-C_3_N_4_, AgBr, Ag/AgBr, and g-C_3_N_4_/Ag/AgBr as photocatalysts in 30 min, within which tetracycline hydrochloride has degraded by 57.5% with g-C_3_N_4_/Ag/AgBr-8% as the photocatalyst. Compared with AgBr (30.0%), Ag/AgBr (35.0%), and g-C_3_N_4_ (8.9%), it can be seen that the photocatalytic activity of g-C_3_N_4_/Ag/AgBr sample has been significantly improved after constructing heterojunctions between g-C_3_N_4_ and Ag/AgBr. Figure 7B shows that g-C_3_N_4_ has the lowest photocatalytic degradation rate, followed by AgBr, and Ag/AgBr has the lowest photocatalytic activity. The kinetic constant of the sample is as high as 0.02764 min^−1^, which is 9.8 times that of g-C_3_N_4_, 2.4 times that of AgBr and 1.9 times that of Ag/AgBr. The photocatalytic activity of the g-C_3_N_4_/Ag/AgBr composite is higher than that of g-C_3_N_4_, AgBr, and Ag/AgBr. Among them, the g-C_3_N_4_/Ag/AgBr-8% sample has the best photocatalytic activity, which also maintains a good stability within six cycles (Figure 7C).

In order to determine the formation and effect of different reactive oxygen species in the photocatalytic process, g-C_3_N_4_/Ag/AgBr-8% is used as the photocatalyst to conduct capture agent experiments, and the results are shown in Figure 7D. In these experiments, ascorbic acid (AA), triethanolamine (TEOA) and isopropanol (IPA) were introduced into the reaction mixture respectively, corresponding to the capture of superoxide radicals (^•^O^2–^), holes (h^+^) and hydroxyl radicals (^•^OH) respectively. It can be seen that although TEOA was added, the decomposition of antibiotics was not significantly hindered. However, in the presence of AA and IPA, the photocatalytic efficiency decreased significantly, especially in the condition where AA was added. This shows that ^•^OH and ^•^O^2–^ are two main reactive oxygen species for the decomposition of tetracycline antibiotics.

### Photocatalytic mechanism

**FIGURE 8:**
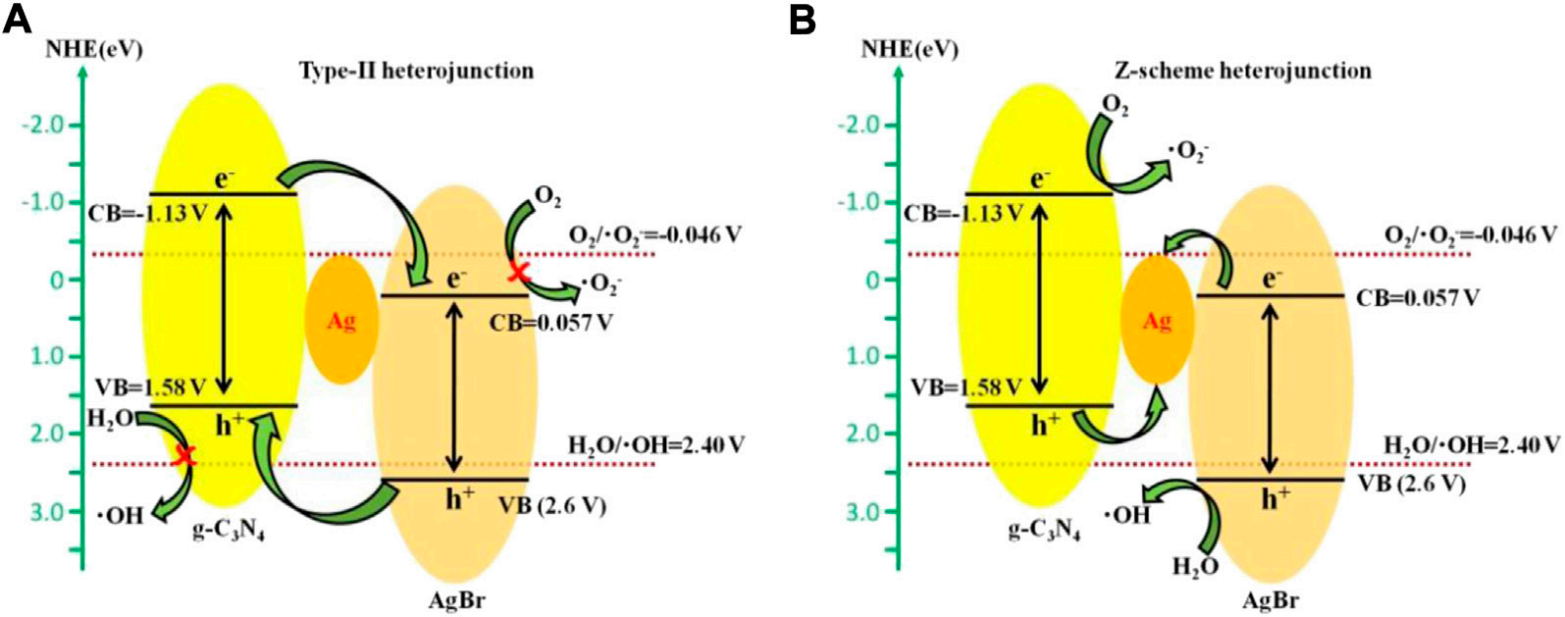
Photocatalytic mechanism of g-C_3_N_4_/Ag/AgBr based on **(A)** type-II heterojunction **(A)** and Z-type heterojunction **(B)**.

According to the energy band positions of AgBr and g-C_3_N_4_, a traditional type-II heterojunction mechanism may be possibly formed between AgBr and g-C_3_N_4_, as is shown in Figure 8A. Under simulated solar light irradiation, both AgBr and g-C_3_N_4_ can be excited to generate electrons and holes. The holes on the VB of AgBr will migrate to that of g-C_3_N_4_, and the electrons on the CB of g-C_3_N_4_ will transfer to AgBr, which is conducive to further participating in the photocatalytic degradation reaction. However, since the conduction band potential of AgBr (0.057 V vs NHE) is lower than the O_2_/^•^O^2–^ potential (−0.046 V vs NHE) (Ye et al., 2012) and the valence band potential of g-C_3_N_4_ (1.58 V vs NHE) is lower than the H_2_O/^•^OH potential (2.40 V vs NHE) (Tian et al., 2015), the electrons accumulated in the AgBr conduction band and the h^+^ accumulated in the g-C_3_N_4_ valence band cannot further generate ^•^O^2–^ or ^•^OH. Therefore, AgBr and g-C_3_N_4_ contact through traditional type-II heterojunctions, ^•^O^2–^ and ^•^OH cannot be formed in the system, and type-II heterojunctions between AgBr and g-C_3_N_4_ are not reasonable. On the contrary, the free radical capture experiment results show that ^•^O^2–^ and ^•^OH are main free radicals involved in the photocatalytic degradation process. Therefore, it can be inferred that photogenerated electrons and holes in the g-C_3_N_4_/Ag/AgBr composite are not transferred through the traditional type-II heterojunction mode. A Z-type heterojunction with silver nanoparticles as bonds is more suitable for explaining the mechanism of the g-C_3_N_4_/Ag/AgBr photocatalysis system. As is shown in Figure 8B, the electrons on the AgBr CB will flow to the Ag nanoparticles under simulated solar light irradiation, which then recombine with the holes from the g-C_3_N_4_ VB, and the photogenerated carriers are effectively separated from the system. The electrons gathering on g-C_3_N_4_ conduction band can fully react with O_2_ to form ^•^O^2–^; the holes on the valence band of AgBr can effectively oxidize H_2_O molecules to generate ^•^OH or directly oxidize organic pollutants. Therefore, it can be inferred that a possible photodegradation enhancement mechanism of g-C_3_N_4_/Ag/AgBr composite is the Z-type heterojunction mechanism.

## Conclusion

Based on the development of new g-C_3_N_4_ heterojunction composite photocatalyst materials, a Z-scheme g-C_3_N_4_/Ag/AgBr photocatalyst was prepared in this work by loading AgBr on g-C_3_N_4_, and then AgBr was reduced to Ag through the photoreduction process. The composite material g-C_3_N_4_/Ag/AgBr shows a good ability in the photocatalysis and degradation of tetracycline hydrochloride under simulated solar light irradiation. Among them, g-C_3_N_4_/Ag/AgBr-8% shows the best photocatalytic degradation ability for tetracycline hydrochloride, and its photocatalytic degradation kinetic constant is as high as 0.02764 min^−1^. The photocatalytic activity of g-C_3_N_4_/Ag/AgBr-8% is 9.8 times that of g-C_3_N_4_ and 1.48 times that of AgBr. The photocurrent response and PL spectra indicate that g-C_3_N_4_/Ag/AgBr composite has a high photo-induced charge separation efficiency. The main active species are ^•^O^2–^ and ^•^OH, which are involved in the photodegradation of organic pollutants. The enhanced photocatalysis mechanism of g-C_3_N_4_/Ag/AgBr composite is mainly attributed to the establishment of a Z-type heterojunction system with silver nanoparticles as the composite center, which makes the energy bands of g-C_3_N_4_ and AgBr match, meanwhile the electrons and holes can shift between their conduction bands and valence bands, effectively reducing the electron hole recombination rate.

## Data Availability

The original contributions presented in the study are included in the article/supplementary material, further inquiries can be directed to the corresponding authors.
